# 

**Published:** 1893-01

**Authors:** 


					﻿Vol. XIII.
JANUARY, 1893.
NO. 1.
JflHE
HOMEOPATHIC PHYSICIAN,
A Monthly Journal of Homoeopathic Materia Medica
and Clinical Medicine.
“He it a freeman whom truth makes free, and all are slaves beside.”
“Seek the Truth: come whence it may, cost what it will."
EDITED AND PUBLISHED BY
WALTER M. JAMES, Ki. ID.
PHILADELPHIA:
No. 1125 SPRUCK STREET.
i/Oots^ljp w:
ALFRED itEATH <fc CO., Sole Agents fob Gbeat Bbitaim,
114 Ebury Street, Eaton Square.
Yearly Subscription, $2.50. invariably in advance. Single numbers, 25 ets.
Entered at the Philadelphia Poet-Offlee as Seoond-Claae Mail Matter.
				

